# Modified Lower Pole Autologous Dermal Sling for Implant Reconstruction in Women Undergoing Immediate Breast Reconstruction after Mastectomy

**DOI:** 10.1155/2016/9301061

**Published:** 2016-10-05

**Authors:** Pankaj G. Roy

**Affiliations:** Nuffield Department of Surgery, John Radcliffe Hospital, Oxford University Hospitals NHS Foundation Trust, Oxford OX3 9DU, UK

## Abstract

*Background*. Autologous dermal sling with wise pattern skin reducing mastectomy allows one-stage implant reconstruction in women with large and ptotic breasts needing mastectomy for cancer or risk reduction. However, this technique is not suitable for women who lack ptosis and also carries risk of T-junction breakdown.* Method*. We have performed one-stage nipple sparing mastectomies with implant reconstruction in 5 women (8 breasts) by modifying the autologous dermal sling approach. All these women had small to moderate breasts with no ptosis or pseudoptosis.* Results*. Three women had bilateral procedures, two underwent bilateral mastectomies simultaneously, and one had contralateral risk reduction surgery a year after the cancer side operation. All women underwent direct to implant reconstruction with implant volumes varying from 320 to 375 cc. There were no implant losses and only one required further surgery to excise the nipple for positive nipple shaves. A low complication rate was encountered in this series with good aesthetic outcome.* Conclusion*. The modified lower pole dermal sling allows direct to implant reconstruction in selected women with small to moderate sized breasts with minimal ptosis. The approach is safe and cost-effective and results in more natural reconstruction with preservation of nipple.

## 1. Introduction

Autologous dermal sling is a safe and effective option to facilitate immediate implant reconstruction for women undergoing mastectomy [1]. This is feasible in women with moderate to large sized ptotic breasts with a need for skin reduction at the time of surgery [1, 2]. The advantages of this approach are as follows: (a) availability of autologous dermal tissue obviates the need for commercially available matrices, making it cost-effective [1], (b) it facilitates one-stage reconstruction if the size of the sling is large enough to accommodate the definitive implant, and (c) the sling has inherent ability to stretch allowing the breast to drop with time, which gives the breast a natural shape as opposed to the more projected and pert breast reconstruction with total submuscular approach; this helps to achieve better symmetry with natural contralateral breast for women undergoing unilateral surgery for breast cancer.

Nipple sparing mastectomy has psychological advantages over skin sparing mastectomy with delayed nipple reconstruction and is oncologically safe in the face of negative retroareolar biopsies in women undergoing mastectomy for cancer [3, 4]. The traditional dermal sling approach is adopted for women with ptotic breasts with nipples well below the ideal position; therefore nipples are often sacrificed, although this can be combined with simultaneous mastopexy to move the nipple on dermal pedicle [5].

We present a modification to the dermal sling approach that would be suitable for women with small to moderate sized breasts with pseudoptosis or minimal ptosis. The additional criteria required for suitability is the distance between inferior areolar edge and inframammary crease should be at least 8 cm. We have performed 8 cases of immediate direct to breast reconstruction with implants after nipple sparing mastectomy.

## 2. Material and Methods

The patients counseled for reconstruction in our unit include the ones with recent diagnosis of breast cancer, cancer naive patients with high-risk family history with/without gene mutation, and patients with previous breast cancer wishing contralateral risk reduction surgery. All patients undergoing risk reduction mastectomies are referred for genetic counseling and assessment. The patients undergoing mastectomy and IBR are counseled for all suitable options of reconstruction.

### 2.1. Indication

The essential criteria for patient selection included patients who were recommended mastectomy (and were suitable for nipple sparing mastectomy) and wished immediate breast reconstruction.

### 2.2. Suitability

The suitability criteria were as follows: small to moderate size breasts (B–D cup) with pseudoptosis or minimal ptosis and distance between inferior areolar edge and inframammary crease more than 8 cm (without stretch).

## 3. Technique

**Figure 1:**
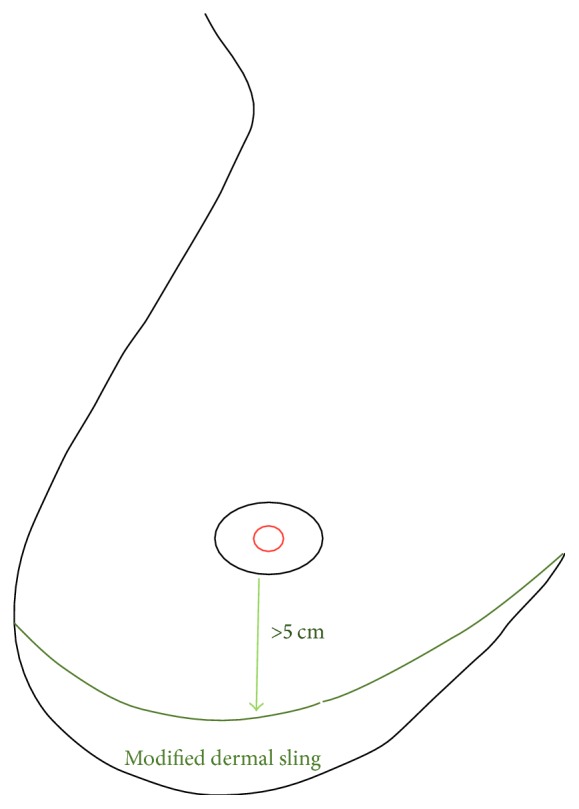
Schematic diagram to show the creation of modified autologous dermal sling (crescent shaped) ensuring the new infra-areolar to inframammary crease distance of at least 5 cm.

The patients were marked preoperatively with a crescent on the lower aspect of the breast ensuring a residual distance of at least 5 cm (ideal distance of 6 cm) between the inferior areolar edge and the inframammary crease (Figure 1). This allowed a dermal sling of at least 3-4 cm in selected cases. The axillary surgery (SLNB, sentinel lymph node biopsy), as and if needed, was performed through a separate scar in the axillary skin crease to isolate the axillary cavity from the implant cavity.

**Figure 2:**
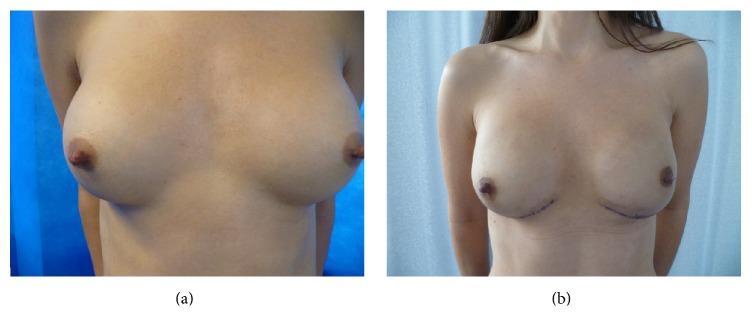
Young lady with small native breasts with bilateral submuscular cosmetic implants in situ, BRCA carrier (Case 1). (a) Pre-op photograph showing lateral displacement of right breast with wide cleavage. (b) Post-op photograph after bilateral nipple sparing mastectomies and one-stage implant reconstruction with modified lower pole dermal sling.

The antibiotics were administered at induction as per local hospital protocol. The incision was made and the dermal sling deepithelialized. The mastectomy was performed carefully ensuring good vascularity of skin flaps and paying attention not to breach the fascia on the chest wall when separating the breast, on the posterior aspect. The retroareolar (nipple) shaves were obtained and sent separately for pathology assessment to ensure the oncological safety of nipple preservation. If the nipple shaves subsequently proved to contain DCIS or invasive cancer, nipple was excised under local anaesthetic at a later date preserving the areola (Figure 2(c)), thus maintaining the shape of the breast.

The dermal sling was raised off the breast ensuring not to breach the fibres forming the inframammary fold. The dermal sling in majority of the patients was not big enough to cover the lateral aspect; therefore we raised the serratus fascia on the lateral aspect in continuation with pectoralis major muscle to define the lateral extent of implant reconstruction. We recommend dividing the pectoralis major fibres along its inferior attachment (and not to detach the muscle along the lateral border) in order to maintain the fascial continuity on the lateral aspect. The inferior detachment was continued medially up to about 5 or 7 o'clock (depending upon the side) to achieve adequate inferomedial fullness; the implant gets covered inferiorly and inferomedially by the dermal sling achieving a more natural shape of the reconstructed breast. After dividing the fibres inferiorly, the pocket was created under the pectoralis major muscle and the dissection is continued laterally continuing under the serratus fascia laterally (and avoid lifting serratus fibres, which is more painful). The pectoralis major fibres were thinned out on the inferomedial attachments on the ribs to allow enough give in the muscle to house a definitive implant.

Once the pocket was created, the wounds were washed out thoroughly with warm saline and 2% chlorhexidine acetate to remove any debris or free fat. The adequacy of the pocket was ensured with an implant sizer and alteration to the pocket made as needed. The medial half of the dermal sling was sutured to the inferior pectoralis edge with sizer in place. This provided an opportunity to assess the final shape and size of the reconstruction before finalizing the implant choice. The wound was washed again, drain(s) was placed as desirable, and the definitive implant was placed in the pocket from the inferolateral aspect. The pocket was sutured completely to ensure total implant coverage. The skin was then sewn in 2 layers. The patients were advised to wear a supportive bra day and night for 2 weeks. Drains were removed once the output is less than 50 mL per day, which varied between 3 and 5 days.

## 4. Results

5 women underwent 8 nipple sparing mastectomies (NSMx) with immediate reconstruction (direct to implant) assisted with autologous dermal sling between 2013 and 2014 using the modification as described above. Three women had bilateral procedures, two underwent bilateral mastectomies simultaneously, and one had risk reduction surgery a year after the cancer side operation. Each case is described below; 5 mastectomies were for risk reduction and 3 for cancer.  Table 1 lists the salient features for all the cases.

**Case 1 (Figure 2).**

33-year-old lady with BRCA2 mutation presented to the clinic for discussion of bilateral risk-reduction surgery. A fit and well lady, employed, had young family and was a nonsmoker. She had small breasts (A cup) and underwent bilateral submuscular augmentation with round 300 cc silicone implants few years prior to presentation (C/D cup). She was very slim built with no options for autologous reconstruction and wished her breasts to be reconstructed to same size. She was counseled for options and decision was made for one-stage implant-based reconstruction with anatomical silicone implants. She had wide cleavage with lateral displacement of right augment.

The nipple sparing mastectomies were performed through inframammary crease incision after preparing a 4 cm dermal sling. The implant pocket was accessed through the inferior aspect, old implants were removed, capsulorrhaphy was performed to adjust the pockets, and Allergan™ high projection, medium height (MX) 370 cc anatomical implants were placed. The breach in implant pocket on the lower aspect of the capsule was buttressed with the dermal sling and wound closed with low-vac drain. The post-op recovery was uneventful with no complications.

**Figure 3:**
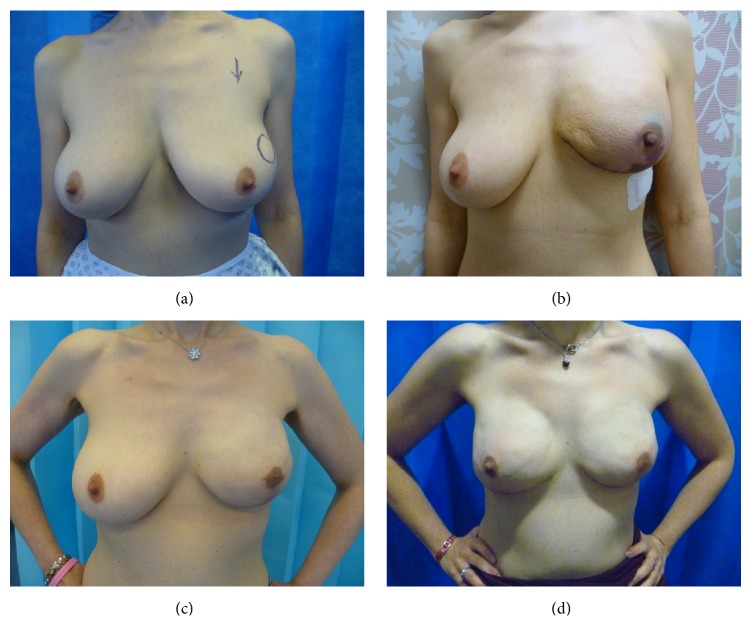
40-year-old lady with left breast cancer (Case 2). (a) Pre-op photograph with skin mark showing the site of cancer in upper outer quadrant of left breast. She has bilateral pseudoptosis. She smoked (1-2 per day) at presentation. (b) Immediate post-op after left NSMx and implant reconstruction. She developed superficial skin necrosis of the skin flap on the inferior aspect that was managed conservatively. The nipple shaves on left side were positive for DCIS. She underwent nipple excision under local anesthetic. (c) The left breast reconstruction after nipple excision. (d) Post-op photograph after right risk-reducing NSMx with one-stage implant reconstruction, 15 months after the surgery on left side. There is a degree of capsular contracture on left side due to radiotherapy.

**Case 2 (Figure 3).**

40-year-old professional with C cup breasts presented with Left breast cancer, which was grade 1, ER (estrogen receptor) positive, Her-2 negative, multifocal. She was recommended mastectomy in view of multifocality. She was fit and well but smoked socially about 1-2 cigarettes every day. She was not a candidate for autologous reconstruction and elected for implant-based reconstruction. She underwent NSMx with SLNB and implant reconstruction (Allergan style 410 MX-370) with a dermal sling. She stopped smoking 3 weeks prior to surgery and was aware of the risks involved. The histology showed extensive unexpected DCIS (ductal carcinoma in situ) measuring 90 mm with multifocal cancer, largest measuring 25 mm grade 2; SLNB showed micrometastasis. The nipple shave was positive for DCIS, so she underwent nipple excision under local anesthetic through an elliptical incision at the nipple base preserving the areola (Figure 3(c)). She developed superficial epidermolysis of left nipple and a patch of superficial skin necrosis inferior to areola, which was managed conservatively with full spontaneous recovery and healing without any scarring.

She received adjuvant chemotherapy, radiotherapy, and tamoxifen. 15 months later, she elected to undergo right (contralateral) risk-reducing mastectomy and underwent the procedure (similar to the one on left,) with an identical implant without any complications. She had developed mild capsular contracture on the left side due to radiotherapy (at review at 2 years following radiotherapy); however this has not yet led to significant asymmetry; therefore no further intervention has been planned to date.

**Case 3 .**

32-year-old lady with B cup breasts presented with right breast cancer, which was grade 2, ER positive, and Her-2 negative. She underwent stand-alone SLNB to stage the axilla, which was negative, prior to neoadjuvant chemotherapy. On subsequent genetic assessment, she tested negative for BRCA mutation but she was still thought to be at high risk of developing second breast cancer. She decided to undergo bilateral mastectomies with implant reconstruction (she was not a candidate for autologous reconstruction due to slim body habitus) and underwent bilateral nipple sparing mastectomies with implant reconstruction (Allergan style 410 MM-320 cc). The histology showed good response to chemotherapy with low volume residual grade 2 invasive ductal cancer over 48 mm associated with DCIS measuring 56 mm. The nipple shaves were negative. She developed small area of epidermolysis of right nipple that healed spontaneously with no aesthetic detriment. She received adjuvant radiotherapy to the right breast and continues on tamoxifen.

**Figure 4:**
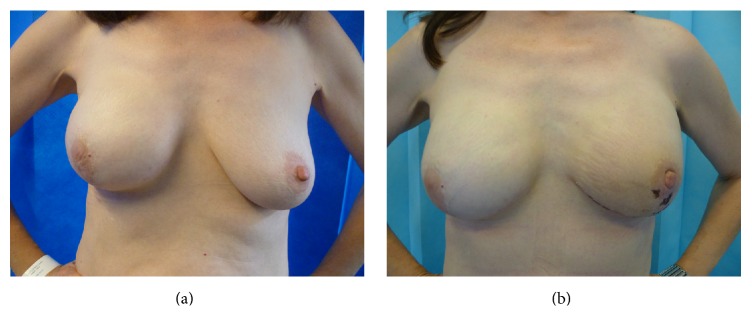
56-year-old lady underwent risk-reducing surgery on left side 3 years after diagnosis of right breast cancer (Case 4). (a) Pre-op photograph showing right breast reconstruction with implant-expander (traditional dermal sling approach). Left breast has lax skin with minimal ptosis. (b) Post-op photograph after left NSMx with implant reconstruction and right breast implant exchange, demonstrating excellent symmetry. She developed small patches of epidermolysis on areola and surrounding skin, which recovered without any active intervention.

**Case 4 (Figure 4).**

56-year-old lady who has had right breast cancer 3 years ago elected to undergo risk-reduction surgery on left side. The right breast cancer was treated with skin-sparing mastectomy (traditional wise pattern, dermal sling approach) and reconstruction with implant-expander (Mentor™ Becker-35) for 4 mm grade 2 invasive ductal cancer, associated with 60 mm high grade DCIS. The cancer was node negative, ER poor, and Her-2 negative. She requested simultaneous right implant exchange for symmetry and to remove the Becker port.

She underwent nipple sparing mastectomy and implant reconstruction with Allergan 410 MF-375 cc anatomical silicone implant and contralateral implant exchange. The post-op recovery was uneventful with small patches of superficial skin necrosis (Figure 4(b)) that healed with conservative management and minimal scarring. She was pleased with the aesthetic outcome and symmetry achieved. She was offered right nipple reconstruction but declined.

**Case 5 .**

41-year-old with D cup breasts presented with left breast cancer, which was grade 2 invasive micropapillary cancer, ER positive, Her-2 positive, and positive axillary node on preoperative ultrasound-guided axillary biopsy. She had neoadjuvant chemotherapy with good clinical response and post-op histology showed small foci of residual low volume disease and 13 nodes removed on axillary clearance were free of disease with evidence of chemotherapy induced fibrosis. She had option of autologous reconstruction with abdominal free flap and decided to keep that in reserve depending on the effect of radiotherapy to her reconstructed breast and her decision for contralateral breast surgery. She, therefore, underwent NSMx with reconstruction with Allergan 410MF-375 cc implant with a view to downsize the reconstructed breast. The nipple shaves were negative. She received adjuvant radiotherapy and tamoxifen.

This lady is not satisfied with resultant smaller size of left breast and the feel of the implant and therefore is considering the option of autologous reconstruction despite minimal postradiotherapy changes.

All women underwent direct to implant reconstruction with implant volumes varying from 320 to 375 cc. There were no implant losses and only one required further surgery to excise the nipple for positive nipple shaves. One patient smoked at presentation. A low complication rate was encountered in this series; all (3 cases out of 8) were related to superficial skin necrosis, of either nipple or the skin flap inferior to the nipple. However, none required further intervention and recovered with conservative management. No seromas were observed that required intervention. All but one patient (Case 5) were satisfied with the aesthetic outcome.

## 5. Discussion

Immediate breast reconstruction in women undergoing mastectomy for breast cancer has psychological advantages [6] and is a cost-effective approach in comparison with delayed breast reconstruction [7]. The choice of reconstruction depends on the patient preferences, patient body habitus, their lifestyle, and likely impact and timing of treatment (especially radiotherapy). When compared with autologous reconstruction, implant reconstruction has the advantages of fast recovery, minimal scarring, and morbidity; the disadvantages being the need for revisional surgery, capsular contracture, and inability to match with the natural breast in patients undergoing unilateral surgery. Implants have regained favour over the last decade due to the availability of commercially available animal products (acellular dermal matrices) and synthetic meshes as these allow one-stage reconstruction. In addition, there is evidence to suggest that the risk of capsular contracture could be reduced by use of ADM [8, 9].

The Bostwick technique [10] uses inferior pole dermal sling in women with large ptotic breast undergoing mastectomy with the skin reducing wise pattern skin incision. The large dermal sling (which is often the case) allows coverage of the implant on inferior and lateral aspect and provides a natural shape and feel to the breast. The nipple-areola complex is usually sacrificed with this approach. Although that can be preserved at the same time by autografting [5] or simultaneous mastopexy [11, 12]; this is not routinely practiced.

The availability of biologic matrices or acellular dermal matrices (ADMs) has extended the indications for implant reconstruction and facilitates one-stage implant reconstruction for women with nonptotic breasts. However the cost of the commercially available products can be prohibitive, especially for social health care systems. The vascularity of the sling makes dermal sling a safer option than use of ADM, which is associated with higher risks such as infection, seroma, and implant loss in comparison to autologous approach [13, 14]. No implant loss was observed in the series. Majority of the complications were superficial skin necrosis, which were managed conservatively with spontaneous healing and minimal impact on the aesthetic outcome. There were no significant seromas observed and none required further surgery for complications. In comparison to direct to implant with ADM, there was higher incidence of superficial epidermolysis (37.5%) noticed in our case series, which is in all likelihood due to the length of incision along the inframammary crease thus increasing the risk to the vascularity of the skin flap. However all cases were managed conservatively without any compromise to the integrity of the implant and/or aesthetic outcome.

T-junction breakdown is a frequently encountered complication (as high as 25%) in traditional dermal sling approach [15, 16]; the modification proposed in this article poses less risk of such wound complications thus reducing the risk of implant loss. The other advantages of this technique (over and above the traditional dermal sling) include less visibility of the scars, nipple preservation, and ability to control the nipple position in cases of preexisting nipple asymmetry.

We have used the modification to perform direct to implant reconstruction in small to moderate sized breasts; but this could be adopted for two-stage or implant-expander reconstruction as deemed appropriate by the treating surgeon, particularly if the reconstructed breast is planned to be bigger than the native breast size.

The modification described helps to achieve one-stage implant reconstruction in carefully selected group of women who are recommended mastectomy. This approach provides an additional bullet in the oncoplastic gun; it is cost-effective and truly one-stage as this permits nipple sparing surgery in women, who do not otherwise fulfill the criteria (large ptotic breasts) required for traditional autologous dermal sling approach. All but one patient were satisfied with the aesthetic outcome during the short follow-up varying between 1 and 3 years in our study. Only one patient required further surgery to excise the nipple (for positive shaves) making this approach truly one-stage reconstruction. That makes it cost-effective and reduces the need for multiple hospital visits that is often required for two-stage reconstruction, which is the option offered to this group of women in the absence of availability of ADMs.

## 6. Conclusion

The modified lower pole dermal sling allows implant reconstruction as a one-stage approach in selected group of women with small to moderate sized breasts. The approach is safe and cost-effective and results in more natural reconstruction with preservation of nipple.

## Figures and Tables

**Table 1:** Clinicopathological details of all the cases undergoing nipple sparing mastectomy with implant reconstruction using the modified dermal sling approach.

	Case 1 Right	Case 1 Left	Case 2 Left	Case 2 Right	Case 3 Right	Case 3 Left	Case 4 Left	Case 5 Left
Age	33	33	40	42	32	32	56	41
Indication	Risk-reducing	Risk-reducing	Cancer	Risk-reducing	Cancer	Risk-reducing	Risk-reducing	Cancer
Year of surgery	2013	2013	2013	2014	2014	2014	2013	2014
Active smoker	No	No	Yes	No	No	No	No	No
Bra cup	A/D	A/D	C	C	B	B	C	D
Breast weight at surgery (g)	80	120	380	560	360	320	275	540
^*∗*^Implant size and shape	370MX	370MX	375MF	375MF	320MM	320MM	375MF	375MF
Nipple shave	Negative	Negative	Positive	Negative	Negative	Negative	Negative	Negative
Complications	None	None	Superficial skin necrosis and nipple epidermolysis	None	Nipple epidermolysis	None	Superficial skin necrosis	None
Chemotherapy	N/A	N/A	Adjuvant	N/A	Neoadjuvant	N/A	N/A	Neoadjuvant
Hormone therapy (Tamoxifen)	N/A	N/A	Yes	N/A	Yes	N/A	N/A	Yes
Herceptin therapy	N/A	N/A	No	N/A	No	N/A	N/A	Yes
Tumour grade	N/A	N/A	2	N/A	2	N/A	N/A	2
Lymph node status	N/A	N/A	Micrometastasis	N/A	Negative	N/A	N/A	Positive
Maximum tumour size (mm)	N/A	N/A	90	N/A	56	N/A	N/A	50

^*∗*^The implants used in the series were Allergan style 410.

N/A: not applicable.

MM: Moderate Height Moderate projection.

MF: Moderate Height Full projection.

MX: Moderate Height Extra full projection.
